# Empagliflozin influences blood viscosity and wall shear stress in subjects with type 2 diabetes mellitus compared with incretin-based therapy

**DOI:** 10.1186/s12933-018-0695-y

**Published:** 2018-04-09

**Authors:** Concetta Irace, Francesco Casciaro, Faustina Barbara Scavelli, Rosa Oliverio, Antonio Cutruzzolà, Claudio Cortese, Agostino Gnasso

**Affiliations:** 1 Department of Health Science, Magna Græcia University, Viale Europa, 88100 Catanzaro, Italy; 2 Department of Experimental and Clinical Medicine, Magna Græcia University, Viale Europa, 88100 Catanzaro, Italy; 3 Department of Experimental Medicine and Surgery, Tor Vergata University, Via Orazio Raimondo 18, Rome, Italy

**Keywords:** Empagliflozin, Blood viscosity, Rheology, Shear stress, Intima-plus media thickness

## Abstract

**Background:**

Cardiovascular protection following empagliflozin therapy is not entirely attributable to the glucose lowering effect. Increased hematocrit might influence the shear stress that is the main force acting on the endothelium, regulating its anti-atherogenic function.

**Objective:**

We designed the study with the aim of investigating the effect of empagliflozin on blood viscosity and shear stress in the carotid arteries. A secondary endpoint was the effect of empagliflozin on carotid artery wall thickness.

**Methods:**

The study was a non-randomized, open, prospective cohort study including 35 type 2 diabetic outpatients who were offered empagliflozin or incretin-based therapy (7 liraglutide, 8 sitagliptin) in combination with insulin and metformin. Blood viscosity, shear stress and carotid wall thickness were measured at baseline and at 1 and 3 months of treatment. Blood viscosity was measured with a viscometer, and shear stress was calculated using a validated formula. Intima-media thickness (IMT) of the carotid artery was detected by ultrasound and was measured with dedicated software.

**Results:**

Blood viscosity (4.87 ± 0.57 vs 5.32 ± 0.66 cP, p < 0.02) and shear stress significantly increased in the Empagliflozin group while no change was detected in the Control group (4.66 ± 0.56 vs 4.98 ± 0.73 cP, p = NS). IMT significantly decreased in the Empagliflozin group after 1 and 3 months (baseline: 831 ± 156, 1-month 793 ± 150, 3-month 766 ± 127 μm; p < 0.0001), while in the liraglutide group, IMT significantly decreased only after 3 months (baseline 879 ± 120; 1-month 861 ± 163; 3-month 802 ± 114 μm; p < 0.001). In the sitagliptin group, IMT remained almost unchanged (baseline 901 ± 135; 1-month 902 ± 129; 3-month 880 ± 140 μm; p = NS).

**Conclusions:**

This study is the first to describe a direct effect of empagliflozin on blood viscosity and wall shear stress. Furthermore, IMT was markedly reduced early on in the Empagliflozin group.

## Background

Treatment of diabetes mellitus has been enhanced in recent years by numerous drugs that have improved the control of the disease. Among these drugs, a recent new class, called gliflozin, has drawn the interest of physicians for its important cardiovascular effects. To date, two large cardiovascular outcome studies have been published, the EMPA-REG Outcome study and the CANVAS study, reporting a positive effect on composite primary cardiovascular endpoints. However, the two studied molecules, empagliflozin and canagliflozin, have provided different results, suggesting a drug effect. Indeed, empagliflozin reduced cardiovascular death and all-cause mortality, as well as hospitalization for heart failure, whereas canagliflozin was neutral on death and heart failure [1, 2]. The new medications inhibit the reabsorption of glucose by the kidney, specifically blocking the sodium–glucose co-transporter protein 2 (SGLT2) located in the proximal tubule [3]. Canagliflozin but not empagliflozin has a moderate SGLT1 inhibitor activity, reducing the absorption of glucose in the gastrointestinal tract, where the SGLT1 is mostly located. A small percentage of SGLT1 (10%) is still present in the latter part of renal proximal tubule [4]. Additional advantages have been described, including body weight reduction due to loss of calories through urine and blood pressure reduction due to transient osmotic diuresis and inhibition of the renin–angiotensin system [5]. SGLT2 inhibitors have recently been demonstrated to markedly reduce cardiovascular mortality with highly significant reduction in admissions for heart failure and end-stage renal disease, despite modest effects on long-term glycemic control [1, 2, 6]. The discrepancy between the glucose lowering effect (similar to that of other treatments) and the effect on cardiovascular events (much greater with SGLT2 inhibitors) has recently attracted great interest. While body weight and blood pressure reduction certainly play a role, other pleiotropic effects have been hypothesized. For example, empagliflozin has been reported to reduce aortic stiffness and afterload, thereby ameliorating ventricular function and myocardial oxygenation [5, 7–9].

An effect consistently described during treatment with SGLT2 inhibitors has been the increase in hematocrit and hemoglobin, thereby improving the ability to transport blood oxygen [10].

Hematocrit is an important determinant of blood viscosity which, in turn, influences blood flow and the development of hemodynamic forces. Wall shear stress (WSS), that is the frictional force acting on the endothelium, is directly proportional to blood viscosity and flow velocity and is inversely proportional to arterial diameter [11]. WSS regulates the release of vasoactive substances by the endothelium, thereby ensuring oxygen supply to the tissues as needed [12]. In addition, WSS is also associated with changes in the arterial wall, including intima-media thickness (IMT) [13].

Keeping this in mind, we designed the present study to test whether treatment with SGLT2 inhibitors was able to influence blood viscosity and WSS. As a secondary endpoint, we tested the effect of empagliflozin on IMT in the carotid arteries of patients with type 2 diabetes mellitus (T2DM) with medium-term follow-up.

## Methods

### Subjects and study design

This report describes a non-randomized, open, prospective cohort, and exploratory study including consecutive subjects with T2DM who were suggested to undergo empagliflozin or incretin-based therapy in combination with insulin ± metformin (background therapy). In these patients, Italian national guidelines recommend the addition of a further glucose-lowering drug if HbA1c is not in the target range. The choice of the drug depended on the patient’s characteristics (fasting and post-prandial glycaemia, body weight, blood pressure, side effects or contraindications) and patient’s preferences (oral vs injectable therapy) [14]. In the present cohort study, the patients received therapy according to these guidelines, and those who were prescribed empagliflozin and incretin-based therapy were asked to participate. The study was approved by the independent local Ethical Committee ‘Calabria Area Centro’ (Protocol Number 105-2016) and was conducted according to ethical guidelines of the Declaration of Helsinki. Before enrollment in the study, the protocol was clearly explained, and the eligible subjects signed the informed consent. Inclusion criteria were age ≥ 18 and < 75, type 2 diabetes, HbA1c > 7% and < 9.5%, current therapy with insulin ± metformin, indication for empagliflozin or incretin-based therapy (GLP-1 analogue or DPP-4 inhibitor). Exclusion criteria were type 1 diabetes, previous treatment with or contraindication to gliflozin or incretin-based therapy, and flow-disturbing stenoses of the carotid arteries.

All subjects underwent three visits: baseline, 1- and 3-month (follow-up visit) after start of therapy. At the baseline visit, subjects were prescribed empagliflozin 10 mg OD or incretin therapy at the starting dose and in accordance with treatment guidelines for T2DM. At the second visit, drugs were titrated as needed. Titration was based on self-monitoring blood glucose (SMBG), and fasting plasma glucose (FPG). If applicable, background glucose-lowering therapy was not changed during the study. Vascular study and shear stress measurement were performed at each visit. A venous blood sample for blood viscosity, HbA1c, FPG, and lipids was collected at baseline and at the 3-month visit. FPG was measured at the 1-month visit. Researchers performing the vascular study and viscosity measurements were blinded with respect to therapy.

Each subject underwent a complete clinical examination during the study. Body weight, heart rate, waist circumference and blood pressure were measured, and concomitant medications and comorbidities were recorded. Subjects were defined as ‘hypertensive’ if they were taking anti-hypertensive drugs or if systolic blood pressure (SBP) and/or diastolic blood pressure (DBP) were respectively > 140/90 mmHg, ‘obese’ if body mass index (BMI) was ≥ 30 kg/m^2^, ‘smokers’ if they had smoked regularly during the previous 12 months, and ‘hyperlipidemic’ if they were taking lipid lowering agents or if they had total cholesterol and/or triglycerides above the targets suggested for diabetes.

### Blood analyses

FPG, HbA1c, and lipids [total cholesterol (chol), high-density lipoprotein (HDL-chol), low-density lipoprotein (LDL-chol) and triglycerides] were measured. Subjects were asked to fast for at least 8 h prior to blood draws and to avoid caffeine and tobacco. Subjects were asked to inject basal insulin the evening before the study and not to take other hypoglycemic drugs the morning of the study. Serum lipids and FPG were measured with commercially available kits. HbA1c was measured with high-performance liquid chromatography aligned with DCCT.

Viscosity measurement was performed the same day of the vascular study. Blood and plasma viscosity were measured within 2 h following blood draws and after adding heparin (35 U ml). Viscosity was measured at 37 °C with a cone-plate viscometer (Well-Brookfield DV-III, Middleboro, MA, US) equipped with a cp-40 spindle. Blood viscosity was evaluated at different shear rates (45, 90, 225/s). Plasma viscosity was measured at shear rate of 225/s. Micro-hematocrit was also measured without correction for plasma trapping. In our laboratory, the coefficient of variation for blood and plasma viscosity was < 3% (2% at shear rate 225/s) and ~ 1% for micro-hematocrit [11].

### Echo-Doppler

All subjects underwent evaluation of carotid arteries at baseline, 1- and 3-month visits. The study was performed using an echo-Doppler Philips HD 11XE (Royal Philips Electronics, the Netherlands) equipped with a 12–3 MHz linear array, steerable pulsed wave Doppler, and simultaneous ECG recording, before blood draws in a temperature-controlled (24 °C) room. The single expert sonographer performing the study was blind to ongoing hypoglycemic treatment. A preliminary scan was used to exclude stenosis > 50% in the extracranial carotid arteries (common, internal and external carotid artery, and carotid bulb), defined as peak systolic velocity of the internal carotid artery > 140 cm/s. Next, the 1 cm of the common carotid artery (CCA) proximal to the bulb was examined in three different projections (anterior, lateral, and posterior) at the R-wave of the cardiac cycle in order to measure IMT in micron (μm) and internal diameter (ID) at the R wave and T wave, as previously reported [11]. IMT was defined as the distance between the leading edge of the lumen-intima interface and the inner edge of the media-adventitia interface of the CCA far wall. IMT was measured off-line using dedicated software (Autodesk^®^ Design Review, BSA Italy) [15]. To estimate the intra-operator reproducibility of the wall thickness evaluation, 10 subjects were studied twice and the coefficient of correlation was r = 0.98. CCA ID was defined as the distance between the leading edge of the intima-lumen interface of the near wall and the leading edge of the lumen-intima interface of the far wall. Blood flow velocities [systolic peak velocity (SPV), end diastolic velocity (EDV), and mean velocity (MV)] of the CCA were measured automatically by the instrument as the means of three cardiac cycles.

Peak WSS (τ_P_) and mean WSS (τ_M_) were calculated based on blood viscosity at shear rate 225/s, blood flow velocity and ID using the following formulas:

$${\text{CCA}}\tau_{\text{P}} \left( {{\text{dyne}}/{\text{cm}}^{ 2} } \right)\, = \, 4 {\upeta \text{SPV}}/{\text{IDT}}$$

$${\text{CCA}}\tau_{\text{M}} \left( {{\text{dyne}}/{\text{cm}}^{ 2} } \right)\, = \, 4 {\upeta \text{MV}}/{\text{IDR}}$$

where η is blood viscosity measured in poise, SPV and MV in cm/s, and ID in cm. CVs of τ_P_ and τ_M_ in our laboratory were 3 and 2%, respectively [11].

### Statistical analyses

Analyses were performed using SPSS 23 for Macintosh. Subjects were divided into two groups: empagliflozin plus background therapy (*Empagliflozin group*), and incretin-activator plus background therapy (*Control group*). Subjects assigned to the Control group were further divided into those who were taking liraglutide and those who were taking sitagliptin in order to evaluate drug-related differences. Variables not normally distributed were waist circumference, triglycerides, plasma and blood viscosity at shear rate 225, 90, 45/s. These variables were log-transformed when applicable or non-parametric test were performed. Right and left peak and mean WSS, and IMT were grouped for statistical analyses. The *t*-*test* for paired data were used to compare continuous variables measured at baseline and follow-up visit. The *t*-*test* for unpaired data was used to compare variables between the Empagliflozin and Control groups. The *Chi square* test was used to compare percentages between the two groups. Analysis of Variance (ANOVA) and Bonferroni post hoc tests were used to evaluate differences among subjects in the three groups (Empagliflozin, Sitagliptin, and Liraglutide). The General Linear Model for repeated measures was applied to evaluate differences among variables detected at three observation-times (baseline, 1-month, and follow-up) in the Empagliflozin and Control groups. The Greenhouse–Geisser correction was applied when the assumption of sphericity was violated.

## Results

Thirty-five subjects who met inclusion and exclusion criteria were enrolled in the study. Twenty were given empagliflozin, and 15 were given incretin-based therapy (7 liraglutide and 8 sitagliptin) as add-on therapy. Overall age, disease duration and HbA1c were 59 ± 8, 16 ± 10 years, and 8.5 ± 1.1% (mean ± SD), respectively. Only two subjects (one in each group) among those enrolled in the study had a previous positive history for coronary heart disease. Thirty-four subjects were hypertensive, and mean disease duration was 5 ± 3 years. Twenty-one were taking RAAS (renin–angiotensin–aldosterone system) inhibitors, and 13 were taking RAAS inhibitors plus diuretic. The prevalence of subjects taking one or two drugs was comparable between the Empagliflozin and Control groups. Thirty-three subjects were hyperlipidemic and were all taking statins. No other vasoactive drugs or supplements were taken. Two subjects with coronary artery disease were taking antiplatelet medication. At baseline, the mean daily insulin dose was 43 ± 20 U in the Empagliflozin group and 39 ± 26 U in the Control group. At the follow-up visit, or end of the study, the mean insulin dose injected per day was 39 ± 17 and 36 ± 23 U in the empagliflozin and the Control group, respectively. The difference was not statistically significant between groups or within groups (either at baseline or end of the study). The percentage of subjects who were taking metformin at baseline was 65% in the Empagliflozin group and 86% in the Control group. All subjects on metformin were taking the maximal tolerated dose and were asked not to modify their therapy during the study. Subjects who were not taking metformin had a history of intolerance to the drug. The starting dose of the new drug was suggested according to drug indications and clinical judgment. Empagliflozin was prescribed at the starting dose of 10 mg OD. The dose was up titrated to 25 mg OD in 13 subjects (65%) at 1-month visit based on FPG and SMBG. Among control subjects, 8 (53%) were prescribed sitagliptin and 7 (47%) liraglutide as add-on therapy. Sitagliptin was prescribed at the dose of 100 mg OD. Liraglutide was prescribed at a starting dose of 0.6 mg OD and was up-titrated to 1.2 mg OD in all subjects after 1-week treatment. Liraglutide was further up-titrated to 1.8 mg OD in four subjects at the 1-month visit based on FPG and SMBG.

Table 1 shows age, gender, disease duration, smoking habits and clinical characteristics of subjects according to therapy. No statistically significant differences were detected between Empagliflozin and Control groups, and no statistically significant difference was found when subjects were divided in three groups (Empagliflozin, Sitagliptin and Liraglutide).

**Table 1:** Age, gender, disease duration, smoking habit and clinical characteristics of subjects according to therapy

	Empagliflozin group	Control group
Number	20	15
Age (years)	58 ± 9	60 ± 7
Males (%)	75	80
Disease duration (years)	15 ± 9	17 ± 10
Obesity (%)	50	40
Hypertension (%)	93	100
Hyperlipidemia (%)	90	93
Smoking habit (%)	30	33

Data are expressed as the mean ± SD or percentage

Biochemical and clinical variables measured at baseline and at 3-month visit were compared and results are shown in Table 2. Body weight, BMI, waist circumference, FPG and HbA1c significantly decreased and HDL-cholesterol significantly increased after 3 months in the Empagliflozin group, while FPG, HbA1c and total cholesterol significantly decreased in the Control group. We again divided control subjects into two groups and compared variables measured at baseline and follow-up visit. In the Liraglutide group, no statistically significant difference was detected between two visits. In the Sitagliptin group, FPG and total-cholesterol significantly decreased (FPG baseline 9.1 ± 0.4 and 3-month 7.4 ± 0.6 mmol/L, p < 0.01; total-cholesterol baseline 4.7 ± 1.8 and 3-month 4.1 ± 1.4 mmol/L, p < 0.01). We also compared baseline and follow-up data between the Empagliflozin and Control groups and found no statistically significant difference except for heart rate, that was significantly higher in the Empagliflozin group at baseline (p = 0.01).

**Table 2:** Biochemical and clinical variables measured at baseline and 3-month in subjects according to therapy

	Empagliflozin group (N = 20)		Control group (N = 15)	
	Baseline	3-month	Baseline	3-month
Body weight (kg)	89 ± 14	85 ± 13*	80 ± 8	79 ± 10
BMI (kg/m^2^)	30 ± 4	29 ± 4*	27 ± 3	26 ± 3
Waist (cm)	107 ± 10	105 ± 10*	102 ± 7	100 ± 8
SBP (mmHg)	141 ± 14	136 ± 16	136 ± 23	135 ± 12
DBP (mmHg)	86 ± 11	84 ± 10	78 ± 8	79 ± 9
HR (bpm)	75 ± 11^#^	69 ± 17	62 ± 6	69 ± 9
FPG (mmol/L)	10.3 ± 3.1	7.9 ± 1.1*	9.7 ± 1.7	8.1 ± 0.9*
HbA1c (%)	8.4 ± 0.7	7.6 ± 0.9*	8.3 ± 0.7	7.7 ± 0.9^^^
Total-chol (mmol/L)	4.09 ± 0.91	4.12 ± 0.98	4.51 ± 1.40	4.17 ± 1.17*
HDL-chol (mmol/L)	1.11 ± 0.34	1.19 ± 0.31*	0.98 ± 0.31	1.04 ± 0.28
LDL-chol (mmol/L)	2.20 ± 0.78	2.28 ± 0.91	2.31 ± 1.17	2.25 ± 0.88
Triglycerides (mmol/L)	1.76 ± 1.18	1.45 ± 0.67	2.13 ± 1.55	1.88 ± 1.29

Data are expressed as the mean ± SD; *t*-test for paired data * p < 0.002, ^^^ p < 0.02 vs baseline; *t*-test for unpaired data ^#^ p = 0.01 vs Control group

Blood viscosity at shear rate 45, 90, 225/s significantly increased during empagliflozin treatment, while no significant change was detected in the Control group, even when control subjects were divided between those taking sitagliptin or liraglutide. Plasma viscosity was unchanged in all subjects throughout the study (Table 3). Blood viscosity at all shear rates was similar in Empagliflozin and Control groups at baseline, however, it was significantly higher at 1 and 3 months in the Empagliflozin than in the Control group.

**Table 3:** Plasma and blood viscosity measured at baseline, 1-month and 3-month in subjects according to therapy

	Empagliflozin group (N = 20)			Control group (N = 15)		
	Baseline	1-month	3-month	Baseline	1-month	3-month
Plasma viscosity	1.48 ± 0.09	1.49 ± 0.11	1.52 ± 0.10	1.47 ± 0.10	1.41 ± 0.06	1.46 ± 0.03
Blood viscosity (45/s)	6.80 ± 0.98	7.47 ± 1.79^^^	7.53 ± 1.05*^^^	6.59 ± 0.88	6.41 ± 0.69	6.35 ± 0.82
Blood viscosity (90/s)	5.77 ± 0.72	6.11 ± 1.10^^^	6.29 ± 0.81*^^^	5.51 ± 0.69	5.35 ± 0.57	5.35 ± 0.64
Blood viscosity (225/s)	4.87 ± 0.57	5.19 ± 0.75^^^	5.32 ± 0.66*^^^	4.66 ± 0.56	4.53 ± 0.50	4.98 ± 0.73
Hematocrit (%)	46.5 ± 3.7	48.2 ± 3.8^^^	49.4 ± 3.8^#^^	45.0 ± 4.3	44.0 ± 4.5	43.9 ± 4.5

Data are expressed as the mean ± SD; * p < 0.02; ^#^ p < 0.0001. General linear model for repeated measures; ^^^ p < 0.01, *t*-test for unpaired data vs Control group

**Fig. 1:**
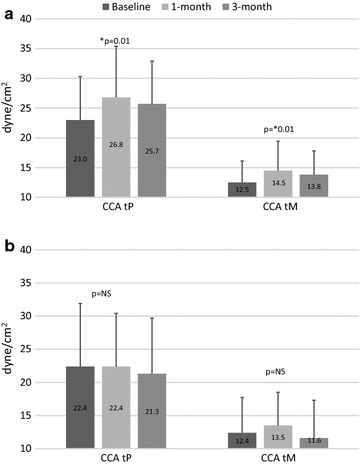
Common carotid artery peak and mean wall shear stress in the Empagliflozin group (**a**) and the Control group (**b**). *p for trend. Bonferroni post hoc analysis: τ_P_ baseline vs 1- and 3-month p = 0.03; τ_M_ baseline vs 1-month p = 0.04, baseline vs 3-month < 0.01

Figure 1 shows CCA peak and mean WSS calculated at each observational point in the Empagliflozin and Control groups. As displayed, shear stress significantly increased in the Empagliflozin group (panel a) but remained stable in the Control group (panel b). Post-hoc analysis revealed a statistically significant difference between baseline and 1-month, and baseline and 3-month peak and mean WSS in the Empagliflozin group. The results were similar when right and left side were analyzed separately. As an example, we reported right and left peak WSS: Empagliflozin group right CCA baseline 23.9 ± 7.9 dyne/cm^2^, 1-month 26.8 ± 8.3 dyne/cm^2^, 3-month 25.6 ± 7.2 dyne/cm^2^, p < 0.01; Empagliflozin group left CCA baseline 25.9 ± 8.1 dyne/cm^2^, 1-month 28.6 ± 9.5 dyne/cm^2^, 3-month 29.8 ± 9.7 dyne/cm^2^, p < 0.01; Control group right CCA baseline 22.7 ± 9.4 dyne/cm^2^, 1-month 22.0 ± 7.3 dyne/cm^2^, 3-month 21.5 ± 7.5 dyne/cm^2^, p = NS; Control group left CCA baseline 21.8 ± 6.5 dyne/cm^2^, 1-month 21.0 ± 4.7 dyne/cm^2^, 3-month 23.2 ± 9.8 dyne/cm^2^, p = NS. Furthermore, peak WSS measured after 1- and 3-month treatment, and mean WSS measured after 3-month treatment were significantly higher in the Empagliflozin group than in the Control group (all p < 0.05). Again, when control subjects were divided according to treatment (liraglutide and sitagliptin), no statistically significant difference was detected among three visits when WSS (peak and mean) were compared. Table 4 shows vascular parameters (right and left common carotid artery grouped) at the three study visits. In the Empagliflozin group, diameter as well as IMT significantly decreased during the three-month period. In patients taking empagliflozin, IMT decreased already after 1-month treatment compared with the Control group, where a statistically significant difference was detected only between baseline and the end of study. When control subjects were divided according to treatment, IMT decreased significantly in the Liraglutide group but not in the Sitagliptin group: Liraglutide, baseline 879 ± 120; 1-month 861 ± 163; 3-month 802 ± 114 μm; p < 0.001; Sitagliptin baseline 901 ± 135; 1-month 902 ± 129; 3-month 880 ± 140 μm; p = NS.

**Table 4:** Mean common carotid artery IMT, internal diameter (ID), and velocities (SPV: systolic peak velocity; EDV: end diastolic velocity; MV: mean velocity) measured at baseline, 1-month visit and 3-month visit

	Empagliflozin group				Control group			
	Baseline	1-month	3-month	p	Baseline	1-month	3-month	p
Number	40	40	40	–	30	30	30	–
ID R (mm)	5.9 ± 0.9^¥^	5.8 ± 0.8	5.8 ± 0.8	0.02	6.0 ± 0.8	5.9 ± 0.8	6.0 ± 0.7	NS
IDT (mm)	6.5 ± 0.9^¥^	6.3 ± 0.8	6.3 ± 0.9	0.02	6.6 ± 0.7	6.5 ± 0.9	6.6 ± 0.8	NS
Mean IMT (μm)	831 ± 156^^^*	793 ± 150	766 ± 127	0.0001	890 ± 146^§^	881 ± 160	841 ± 109	0.01
SPV (cm/s)	81 ± 18	83.6 ± 18	80 ± 13	NS	77 ± 18	77 ± 17	80 ± 18	NS
EDV (cm/s)	25 ± 6	25 ± 6	24 ± 4	NS	24 ± 6	27 ± 5	24 ± 8	NS
MV (cm/s)	41 ± 8	41 ± 4	39 ± 6	NS	38 ± 7	41 ± 2	40 ± 10	NS

Bonferroni post hoc test: * vs 1-month p = 0.03; ^^^ vs 3-month p = 0.002; ^§^ vs 3-month p = 0.03; ^¥^ vs 1-month p = 0.04

Mean IMT results were comparable when right and left CCA were analyzed separately in the Empagliflozin group. In the Control group, the difference among the three observation times was significantly different only for the left side. Empagliflozin group *right* IMT: baseline 815 ± 152 μm, 1-month 785 ± 143 μm, 3-month 755 ± 117 μm, p = 0.03; *left* IMT: baseline 850 ± 177 μm, 1-month 812 ± 172 μm, 3-month 786 ± 152 μm, p < 0.01. Control group *right* IMT: baseline 843 ± 171 μm, 1-month 841 ± 163 μm, 3-month 797 ± 165 μm, p = NS. Control group *left* IMT: baseline 936 ± 105 μm, 1-month 922 ± 155 μm, 3-month 839 ± 114 μm, p < 0.01. We obtained similar results when control subjects were analyzed separately. No significant change in arterial diameter was detected in the Control group.

## Discussion

The present study was designed with the aim of evaluating the effect on blood viscosity and common carotid wall shear stress of empagliflozin when added to background therapy with insulin ± metformin. As an additional aim, knowing the close relationship between shear stress and artery wall thickness, we also evaluated the effect of empagliflozin on carotid intima-media thickness. The study demonstrates, for the first time to our knowledge, that empagliflozin significantly increased blood viscosity after 1 and 3 months of therapy, while incretin-based therapy given in the Control group did not. Common carotid artery diameter significantly decreased after empagliflozin treatment, but not after incretin therapy. Blood flow velocity remained unchanged with both treatments. As a consequence, common carotid wall shear stress significantly increased in subjects taking empagliflozin and remained stable in those taking incretin. As an additional finding, mean IMT decreased in controls (though limited to the Liraglutide group) but did so more markedly and earlier in patients taking empagliflozin.

The effect of SGLT2 inhibitors on hematocrit has been described, but its importance has been questioned. Some authors reported this phenomenon as a potentially negative effect, while others have simply described the finding without comment [16–19]. The relationship between hematocrit, diabetes mellitus and cardiovascular diseases is very complex [20]. Increased hematocrit level seems to have negative impact on glucose metabolism, that is, an increased risk of developing diabetes or prediabetes [21, 22]. However, we have recently demonstrated that a sudden reduction of hematocrit and blood viscosity, occurring following blood donation, has no effect on insulin resistance in healthy subjects [23]. Furthermore, Salazar Vázquez et al. even demonstrated cardiovascular benefits associated with moderate increases in blood viscosity [24]. As a matter of fact, we do not know the optimal range of hematocrit or blood viscosity for metabolic and vascular functions. Consequently, we do not know whether a change in hematocrit has positive or negative effects. However, we do know that SGLT2 inhibitors have protective cardiovascular effects while reducing plasma volume and increasing hematocrit through the increase of diuresis and the excretion of urinary sodium [25–27]. Our results are in line with these findings, and further demonstrate for the first time that the increased hematocrit observed in other studies do actually translate, as one would anticipate, into increased blood viscosity. Our data are also in line with an analysis recently published on EMPA-REG Outcome Trial, showing that changes in markers of plasma volume were the most important mediators of the reduction in risk of CV death with empagliflozin vs placebo [27].

The present study was of short duration, raising doubt that the observed effect on blood viscosity could be reduced or could disappear over time. However, a recent analysis reported early and sustained long-term effect of treatment with empagliflozin on estimated plasma volume. Although long-term studies are necessary, this could suggest a sustained improvement in hemodynamic changes [28].

In the present study, blood flow velocity remained stable during the observation period, both in patients taking empagliflozin or incretin, and carotid artery diameter did not change in the Incretin group while slightly decreasing in empagliflozin patients. The observed increase in wall shear stress in patients taking empagliflozin was mainly sustained by increased blood viscosity. It is known that atherosclerotic lesions developed in arteries presenting low wall shear stress, and plaque thickness decreased when wall shear stress increased [29–31]. Again, a range or threshold value for common carotid wall shear stress has not been established, but the available evidence suggests a potentially beneficial effect of an increase in wall shear stress of the entity observed in the present study [15].

IMT is a reliable marker of early atherosclerosis, is an established risk factor for incident cardiovascular acute events, and is used to evaluate the global cardiovascular risk [32]. Carotid IMT has been frequently monitored in interventional studies to evaluate the efficacy of several drugs in terms of cardiovascular protection [33–35]. IMT was inversely and strongly associated with wall shear stress [11]. In this study, we demonstrated, in healthy male subjects, that a difference in ~ 5 dynes/cm^2^ was associated with roughly a 4.5% difference in IMT. In the present study, peak wall shear stress in the left common carotid artery increased by almost 5 dynes/cm^2^ after 3-month therapy with empagliflozin, while mean IMT was reduced by 7.9%. These data, taken together, are impressive and intriguing. The reduction of IMT was marked and already evident after only 1-month therapy. IMT is considered a marker of structural modification of the vessel wall and as such is quite stable over time. The clinical significance of this reduction might seem questionable. However, the IMT reduction observed in the present study, in a period of only 3 months, was higher than the expected annual progression in diabetic subjects, thus suggesting possible relevant clinical effects [36]. Lipid-lowering drugs and non-pharmacological treatments have been shown to reduce IMT or slow its progression over relatively long periods of time [37, 38]. However, none of these interventions caused a marked change in carotid hemodynamics. In a previous study of subjects undergoing aortic valve replacement, after a procedure that induced prompt modification of shear stress comparable to that observed in the present study, we found carotid IMT reduction up to 122 μm after 1 month [31]. These data and the present investigation suggest that the arterial wall is very quick to adapt to hemodynamic changes, which should be considered when planning intervention studies. Two ongoing studies will provide important information on the effect of SGLT2 inhibitors on IMT [39, 40].

We have also found a beneficial effect of liraglutide group after 3-month treatment. The effect was less pronounced compared with empagliflozin and was not correlated to blood viscosity and wall shear stress modifications. The incretin-based drugs are new molecules improving not only glycemic control but also influencing cardiovascular risk and early atherosclerosis. As known from the literature, sitagliptin attenuated the progression of carotid wall thickening although it did not reduce cardiovascular events and mortality [41], and liraglutide reduced cardiovascular mortality [42, 43]. In the new era of antidiabetic treatment, the reduction of glycemic parameters such as HbA1c and FPG or post-prandial glycemia needs to be paralleled by a positive influence on the prognosis of the disease. Indeed, recent cardiovascular outcome trials proceeded in this direction. However, the interesting point of our study was that gliflozin worked also by influencing blood properties such as viscosity, and hemodynamic forces such as shear stress. Incretin-based therapy likely works by influencing different pathways. Therefore, one cannot exclude the notion that the two drugs in combination might work in synergistically.

The present study has some limitations, but two are probably of major importance: The first limitation was the design of the study. Indeed, it was a non-randomized and open study. However, in an attempt to reduce the impact of the open design, we planned that the operator performing echo-Doppler examination was totally blinded to treatment as was the technician performing viscosity analyses. Furthermore, even the physician prescribing the therapy was blind to IMT evaluation and blood viscosity measurement. The second limitation was the low number of subjects enrolled in the study. The sample size was calculated based on expected change in blood viscosity. Therefore, we believe the number of patients calculated to be sufficient for the purpose of the present study might be insufficient for different analyses.

The choice to prescribe additional drugs following guidelines might seem to be a further limitation of the study. However, national guidelines that are in line with international guidelines and frequently updated suggest a wide choice of drugs as add-on therapy, not limiting the physician’s therapeutic choice [14].

## Conclusions

The present study first demonstrated that the SGLT2 inhibitor empagliflozin caused significant increases in hematocrit and blood viscosity. Common carotid wall shear stress was consequently increased and, probably as an adaptive phenomenon, IMT was reduced. The study offers the opportunity to investigate the additional effects of gliflozin that may contribute to the beneficial cardiovascular effects of this drug.

## Abbreviations

SGLT2: sodium–glucose co-transporter protein 2
WSS: wall shear stress
IMT: intima-media thickness
T2DM: type 2 diabetes mellitus
HbA1c: glycated hemoglobin
GLP-1: glucagon-like peptide 1
DPP-4: dipeptidyl peptidase-4
FPG: fasting plasma glucose
SBP: systolic blood pressure
DBP: diastolic blood pressure
BMI: body mass index
LDL: low-density lipoprotein
HDL: high-density lipoprotein
CCA: common carotid artery
ID: internal diameter
SPV: systolic peak velocity
EDV: end diastolic velocity
MV: mean velocity
Peak WSS (τ_P_): peak wall shear stress
WSS (τ_M_): mean wall shear stress
η: blood viscosity
OD: once daily
SMBG: self-monitoring blood glucose
